# Comparative Analysis of Intensive Care Prognosis Scoring Systems and Acute Kidney Injury Scores (AKIN and pRIFLE) in Critically Ill Children

**DOI:** 10.3390/children10030484

**Published:** 2023-03-01

**Authors:** Ayben Leblebici, Gurkan Bozan, Asli Kavaz Tufan, Eylem Kiral, Ebru Kacmaz, Ener Cagri Dinleyici

**Affiliations:** 1 Department of Pediatrics, Faculty of Medicine, Eskisehir Osmangazi University, Eskisehir 26040, Turkey; 2 Pediatric Intensive Care Unit, Faculty of Medicine, Eskisehir Osmangazi University, Eskisehir 26040, Turkey; 3 Department of Pediatric Nephrology, Faculty of Medicine, Eskisehir Osmangazi University, Eskisehir 26040, Turkey

**Keywords:** AKIN, pRIFLE, PRISM III, PIM-2, PELOD-2, pSOFA, pediatric intensive care, Mortality

## Abstract

The development of AKI (acute kidney injury) in critically ill patients in pediatric intensive care units (PICUs) is one of the most important factors affecting mortality. There are scoring modalities used to predict mortality in PICUs. We compared the AKIN (Acute Kidney Injury Network) and pRIFLE (pediatric risk, injury, failure, loss, and end stage) AKI classifications and PICU scoring modalities in this study. Methods: A total of 716 children, whose serum creatinine levels were within the normal limits at the time of admission to the PICU between January 2018 and December 2020, were included. Along with the demographic and clinical variables, AKIN and pRIFLE classifications were recorded at the most advanced stage of AKI. Along with the PIM-2, PRISM III, and PELOD-2 scores, the highest value of the pSOFA score was recorded. Results: According to the pRIFLE and AKIN classifications, 62 (8.7%) patients developed kidney injury, which had a statistically significant effect on mortality. The occurrence of renal injury was found to be statistically strongly and significantly correlated with high PRISM III, PELOD-2, and pSOFA scores. When the stages of kidney injury according to the AKIN criteria were compared with the PRISM III, PELOD 2, and pSOFA scores, a significant difference was found between the patients who did not develop AKI and those who developed stage 1, stage 2, and stage 3 kidney injury. For the PRISM III, PELOD 2, and pSOFA scores, there were no significant differences between the stages according to the AKIN criteria. A substantial difference was discovered between the patients who did not develop AKI and those who were in the risk, injury, and failure plus loss stages according to the pRIFLE criteria. According to the PIM-2 ratio and pRIFLE criteria, there was a statistically significant difference between patients in the injury and failure plus loss stages and those who did not develop AKI. Conclusions: Our study is the first pediatric study to show a substantial correlation between the variables associated with the PICU scoring modalities in critically ill children with AKI. Identifying the risk factors for the development of AKI and planning antimicrobial regimens for patients with favorable prognoses at the time of PICU admission could lower mortality rates.

## 1. Introduction

Acute kidney injury (AKI) in children is a life-threatening clinical condition in which numerous organs and systems are impacted by a rapid decline in the glomerular filtration rate (GFR), accumulation of uremic toxins such as urea and serum creatinine (sCr), and deterioration of kidney structure and function over the course of hours or days. AKI is typically treatable, but in rare cases, it might progress to chronic kidney disease (CKD) [1,2]. The incidence of AKI in patients hospitalized in intensive care units (ICUs) has been reported to range from 3 to 82%, and in pediatric intensive care units (PICUs), it ranges from 8 to 92% [3,4]. In recent years, AKI has emerged as one of the variables that has the most significant unfavorable impact on the clinical trajectory of patients admitted to PICUs [5]. When ICU admission is necessary for a patient with an underlying chronic illness and nephrotoxic treatment, such as sepsis, cancer, hypoxemia, cardiovascular dysfunction, or reduced cardiac output following cardiac surgery, the risk of AKI is raised [6,7].

To diagnose AKI with a single definition, various classifications have been developed. A definition and categorization approach for AKI known as the risk, injury, failure, loss, and end-stage (RIFLE) criteria has been developed, and its application is now widely accepted [8]. In the pediatric RIFLE (pRIFLE) criteria, which were developed by modifying the RIFLE criteria for children, the basis is a lower sCr level in pediatric patients and lower urine output compared to adults [9]. In order to increase the sensitivity of the RIFLE identification system, the “Acute Kidney Injury Network” has developed a classification system called AKIN, which evaluates changes in kidney function within 48 h. The RIFLE and AKIN criteria use changes in sCr value and urine output, independent of etiology [10]. Many different scoring systems have also been created to compare care between various units, to compare the care standards of the same unit over time, to compare between patient groups, and to predict the mortality and morbidity risk of critically ill children followed up and treated in a PICU [11,12]. The Pediatric Mortality Risk Score (PRISM) III and Pediatric Mortality Index (PIM)-2 scores are the two most widely used mortality scoring systems in PICUs [11,12]. Pediatric Sequential Organ Failure Assessment (pSOFA) and Pediatric Logistic Organ Dysfunction-2 (PELOD-2) are the two most widely used scoring systems for organ failure [13,14].

There are no previous publications about the relationship between mortality scoring systems and scores related to AKI. The purpose of this study was to examine whether or not there is a correlation between scores in the PICU among children diagnosed with AKI based on the AKIN and pRIFLE criteria.

## 2. Materials and Methods

This was a single-center, retrospective, cross-sectional study of patients who were admitted to a tertiary PICU. Medical records of the 785 patients, who were aged between 1 month and 18 years and followed up in the PICU between January 2018 and December 2020, were evaluated. The exclusion criteria were high creatinine levels at admission due to any cause. This study was planned in accordance with the Helsinki Declaration decisions, patient rights regulations, and ethical committees, and approval from the ethics committee was obtained. Age, gender, the presence of underlying conditions, all medications, the presence of mechanical ventilation, length of PICU stay, and prognosis were noted from the medical records.

All of the patients’ stages of acute kidney injury were determined using the pRIFLE and AKIN criteria. A total of five groups were formed by evaluating the decrease in GFR and changes in urine output within seven-day periods according to the pRIFLE criteria, in addition to the three stages of “risk,” “injury,” and “failure” according to the severity of AKI, and the two stages of “loss” and “end stage” according to the clinical outcome [8]. Three stages of AKI were defined according to the AKIN criteria by evaluating the increase in sCr levels and changes in urine output within 48 h periods [10].

The PRISM-III score includes systolic blood pressure, heart rate, body temperature, Glasgow coma score, and pupillary reflex, as well as acidosis, pH, pCO_2_, pO_2_, and total CO_2_ for acid–base balance, and serum glucose, potassium, creatinine, blood urea nitrogen, white blood cell count, platelet count, prothrombin time, and activated partial thromboplastin time in biochemical tests. It is an intensive care scoring system that is calculated by evaluating the first 24 h of intensive care unit admission. The PIM-2 score is calculated within the first hour of the patient’s admission to the intensive care unit. The PIM-2 score is calculated with the scores given according to the patient’s systolic blood pressure, pupillary response, FiO_2_ × 100/PaO_2_, baseline gap, need for mechanical ventilation, and hospitalization diagnosis. PELOD-2 and pSOFA scores are organ failure scores in which the highest value is recorded by evaluating the patient every 24 h during their stay in the intensive care unit. In pSOFA, PaO_2_/FiO_2_, SpO_2_/FiO_2_, platelets, bilirubin, mean arterial pressure, inotrope requirement, serum creatinine, and Glasgow coma score are evaluated. In PELOD-2, pupil response, serum lactate level, PaCO_2_, mechanical ventilation need, and white blood cell count are recorded and calculated. The PIM-2 score, which was computed using the data at the time of admission to the PICU, and the PRISM III score, which was derived using the patient’s data in the first 24 h of admission, were noted from the file records. Throughout the course of the PICU stay, the highest PELOD-2 and pSOFA values were determined every 24 h [11,12,13,14].

Statistical analysis: The statistical analyses were evaluated using the Statistical Package for Social Sciences (SPSS 15.0, IBM, United States). None of the continuous variables displayed a normal distribution, which was assessed using histograms, Q–Q plots, and the Shapiro–Wilk test. Continuous variables are presented as median (minimum to maximum) values. Variables that were categorized are displayed as frequency and percentage distributions. Comparisons between two groups and more than two groups were performed using the Mann–Whitney U test, the Kruskal–Wallis test, the chi-square test, or the Fischer’s exact test. The Spearman correlation coefficient test was used for correlations. In the multivariate analysis, the variables that were shown to have a statistically significant impact on mortality and kidney injury outcomes in the univariate analysis were included as covariates using a forward logistic regression model. A *p*-value of <0.05 was considered statistically significant.

## 3. Results

In total, 785 PICU admissions between January 2018 and December 2020 were evaluated. Fifty-nine children with high creatinine levels at admission and ten children with incomplete medical records were excluded from the study. Finally, the medical records of 716 children (346 girls and 370 boys) aged between 1 month and 218 months (median: 47 months) were analyzed. The median length of stay in the PICU was 4 days (0–746 days). Of the 716 children, 29.9% (n = 214) needed mechanical ventilation, 76.6% of the patients who needed mechanical ventilation needed invasive mechanical ventilation, and 23.4% needed non-invasive mechanical ventilation. The most common primary diagnoses for hospitalization in the PICU were a lower respiratory tract infection (24.4%), intoxication (13.9%), and seizures (11.7%). The mortality rate was 8.5% (n = 61). A total of 577 (80.6%) patients had only one primary diagnosis, compared to 119 (16.6%), 16 (2.2%), 3 (0.4%), and 1 (0.1%) who had two, three, four, and five primary diagnoses, respectively. More than half of the patients (n = 394, 55%) had no previous or additional comorbidities at admission. One comorbidity was present in 221 (30.9%) individuals, two were present in 79 (11%), three were present in 20 (2.8%), and four were present in 2 (0.3%). The most prevalent comorbidity was neurological disorders (n = 138; 19.3%), followed by genetic syndromes (n = 57; 8%) and gastrointestinal disorders (n = 52; 7.3%).

According to the pRIFLE and AKIN criteria, 8.7% (n = 62) of the patients had an acute kidney injury. According to the pRIFLE criteria, 17 (2.4%) patients were at risk for kidney injury, while 28 (3.9%) patients had an injury, 16 (2.2%) patients had a failure, and 1 (0.1%) patient had a loss. According to the AKIN criteria, 19 (2.7%) patients were stage 1, 28 (3.9%) patients were stage 2, and 15 (2.1%) patients were stage 3. A strong correlation was found between the pRIFLE and AKIN criteria (correlation coefficient = 0.984, *p* < 0.001). AKI patients were significantly younger (*p* < 0.01), and boys had significantly higher levels (*p* < 0.05) (Table 1). In patients with kidney injury, the number of primary diagnoses per patient (*p* < 0.001) and the number of comorbidities (*p* < 0.001) were found to be higher. In patients with kidney injury, mechanical ventilation (*p* < 0.001) and invasive mechanical ventilation (*p* < 0.001) requirements were significantly higher (Table 1). The length of hospital stay was found to be longer (*p* < 0.001) in patients with kidney injury (Table 1). The PRISM III (*p* < 0.001), PELOD 2 (*p* < 0.001), pSOFA (*p* < 0.001), and PIM 2 (*p* < 0.001) scores were also higher in patients with kidney injury (Table 1). In patients with kidney injury, the usage of antibiotics (*p* < 0.001), antifungals (*p* < 0.001), antiepileptics (*p* < 0.05), steroids (*p* < 0.001), inotropes (*p* < 0.001), proton pump inhibitors (*p* < 0.001), sedative drugs (*p* < 0.001), paracetamol/acetyl salicylic acid (*p* < 0.001), ibuprofen (*p* = 0.001), opioid analgesics (*p* < 0.001), diuretics (*p* < 0.001), antihypertensive medications (*p* < 0.001), antiarrhythmics (*p* < 0.001), ketoprofen (*p* < 0.05), and vasopressin (*p* < 0.001) was found to be significantly higher (Table 1).

In patients with kidney injury, the median number of antibiotics used was significantly higher (*p* 0.001). In patients with kidney injury, however, the rates of using antifungals with antibiotics (*p* 0.001) and antivirals and antifungals together with antibiotics (*p* = 0.015) were significantly higher. In the subgroup analysis of the effect of antibiotics on kidney injury, in patients with kidney injury, carbapenem (*p* < 0.001), macrolide (*p* = 0.003), glycopeptide (*p* < 0.001), beta-lactam (*p* = 0.009), cephalosporin (*p* = 0.014), penicillin (*p* < 0.001), colistin (*p* < 0.001), aminoglycoside (*p* < 0.001), glycylcycline (*p* < 0.001), quinolone (*p* < 0.001), lincosamide (*p* < 0.001), and sulfonamide/cotrimaxazole (*p* < 0.001) usage rates were found to be significantly higher (Table 1). A significant correlation was also found between the number of antibiotics and the pRIFLE stage (r = 0.324, *p* < 0.001) and the AKIN stage (r = 0.324, *p* < 0.001). In total, 47% of the patients who did not survive had kidney injury, compared to only 5% of the patients who did survive (*p* < 0.001). According to the pRIFLE (*p* < 0.001) and AKIN criteria (*p* < 0.001), a significant proportion of patients with mortality were found to have a more advanced stage of AKI (Table 2).

When the effect of continuous variables on mortality was investigated, the mortality rate was higher in patients with a younger age (*p* < 0.001), comorbidities (*p* < 0.001), a longer length of hospital stay (*p* < 0.001), and increased PRISM III (*p* < 0.001), PELOD-2 (*p* < 0.001), pSOFA (*p* < 0.001), and PIM-2 (*p* < 0.001) scores (Table 2). In the logistic regression model analysis performed for mortality, age, number of comorbidities, development of kidney injury, and use of carbapenem, cephalosporin, penicillin, and glycylcycline were found to have a significant effect on mortality. It was found that the pSOFA score was significantly associated with mortality.

### 3.1. AKIN Criteria

According to the AKIN criteria, there was no significant difference in age between patients with and without kidney injury (*p* > 0.05). There was a significant difference between patients with and without kidney injury (*p* < 0.05) and those in stages 2 and 3 (*p* < 0.01). While a significant difference was found between stages 1 and 3 (*p* < 0.05), there was no significant difference between stages 1 and 2 (*p* > 0.05) and between stages 2 and 3 (*p* > 0.05). When the subgroup analysis considered the length of hospital stay, there was a significant difference between patients who did not develop kidney injury and those with stage 1 (*p* < 0.001), stage 2 (*p* < 0.001), and stage 3 (*p* < 0.001) kidney injury according to the AKIN criteria.

According to the AKIN criteria, the median PRISM III score was 1 (0–39) in patients who did not develop kidney injury, 5 (0–40) in kidney injury stage 1 patients, 4 (0–35) in stage 2 patients, and 5 (0–41) in stage 3 patients.

**Figure 1:**
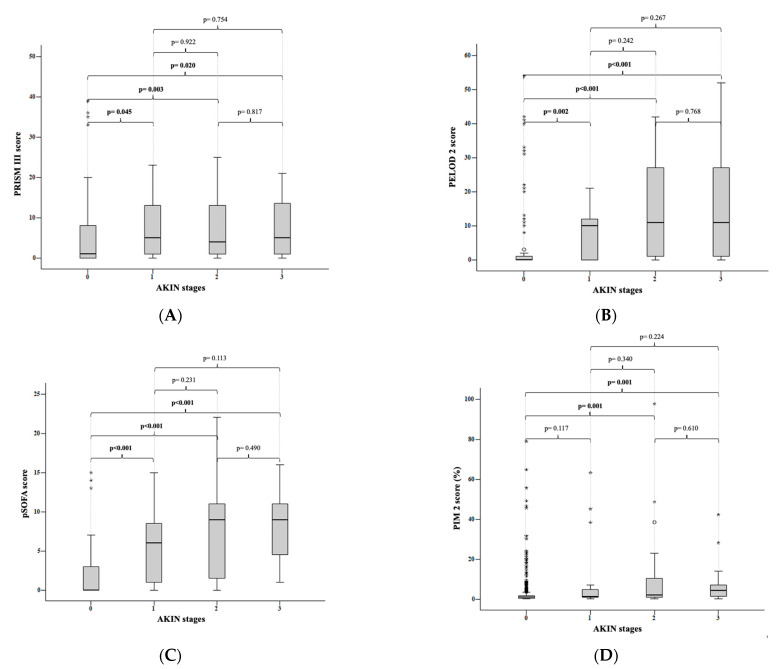
(**A**) AKIN stages and PRISM III scores: The stages of kidney injury according to the AKIN criteria were compared with the PRISM III scores. A significant difference was found between the patients who did not develop kidney injury and those who developed stage 1 (*p* = 0.045), stage 2 (*p* = 0.003), and stage 3 (*p* = 0.020) kidney injury. There was no significant difference between stages 1 and 2 (*p* = 0.922), between stages 1 and 3 (*p* = 0.754), and between stages 2 and 3 (*p* = 0.817). (**B**) AKIN stages and PELOD 2 scores: When the medians of the PELOD 2 score were compared with the stages of kidney injury according to the AKIN criteria, there was a significant difference between patients who did not develop kidney injury and those who developed stage 1 (*p* = 0.002), stage 2 (*p* < 0.001), and stage 3 (*p* < 0.001) kidney injury. There was no significant difference between stage 1 and 2 kidney injuries (*p* = 0.242), between stage 1 and 3 kidney injuries (*p* = 0.267), and between stage 2 and 3 kidney injuries (*p* = 0.768). (**C**) AKIN stages and pSOFA scores: According to the AKIN criteria, there was a statistically significant difference between patients who did not develop kidney injury and those who developed stage 1 (*p* < 0.001), stage 2 (*p* < 0.001), and stage 3 (*p* < 0.001) kidney injury. While there was a statistically significant difference between stages 1 and 2 (*p* = 0.231), there was no statistically significant difference between stages 1 and 3 (*p* = 0.113) and between stages 2 and 3 (*p* = 0.490). (**D**) AKIN stages and PIM 2 scores: According to the AKIN criteria, there was no significant difference between the patients who did not develop renal injury and those who developed stage 1 (*p* = 0.117) kidney injury when the stages of kidney injury were compared with the medians of the PIM-2 score. There was a significant difference between patients without kidney injury and patients with stage 2 (*p* = 0.001) and stage 3 (*p* = 0.001) kidney injury. There was no significant difference between stage 1 and 2 kidney injuries (*p* = 0.340), between stage 1 and 3 kidney injuries (*p* = 0.224), and between stage 2 and 3 kidney injuries (*p* = 0.610).

When the stages of kidney injury according to the AKIN criteria were compared with the PRISM III score, a significant difference was found between the patients who did not develop kidney injury and those who developed stage 1 (*p* = 0.045), stage 2 (*p* = 0.003), and stage 3 (*p* = 0.020) kidney injury. There was no significant difference between stages 1 and 2 (*p* = 0.922), between stages 1 and 3 (*p* = 0.754), and between stages 2 and 3 (*p* = 0.817) (Figure 1A).

According to the AKIN criteria, the median PELOD-2 score for patients without kidney injury was 0 (0–54); for stage 1 patients, it was 10 (0–60); for stage 2 patients, it was 11 (0–42); and for stage 3 patients, it was 11 (0–52). When the medians of the PELOD 2 score were compared with the stages of kidney injury according to the AKIN criteria; there was a significant difference between patients who did not develop kidney injury and those who developed stage 1 (*p* = 0.002), stage 2 (*p* < 0.001), and stage 3 (*p* < 0.001) kidney injury. However, there was no significant difference between stage 1 and 2 kidney injuries (*p* = 0.242), between stage 1 and 3 kidney injuries (*p* = 0.267), and between stage 2 and 3 kidney injuries (*p* = 0.768). This was seen when the medians of the PELOD-2 score were compared according to the stages of kidney injury (Figure 1B).

In the subgroup analysis of the pSOFA scores, the median pSOFA score for patients without renal injury was 0 (0–15), followed by 6 (0–15), 11 (0–42) for stage 1 patients, 11 (0–42) for stage 2 patients, and 11 (0–52) for stage 3 patients. According to the AKIN criteria, there was a statistically significant difference between patients who did not develop kidney injury and those who developed stage 1 (*p* < 0.001), stage 2 (*p* < 0.001), and stage 3 (*p* < 0.001) kidney injury. While there was a statistically significant difference between stages 1 and 2 (*p* = 0.231), there was no statistically significant difference between stages 1 and 3 (*p* = 0.113), and between stages 2 and 3 (*p* = 0.490) (Figure 1C).

When the subgroup analysis considered the PIM-2 score, according to the AKIN criteria, the median PIM-2 score was 1 (0.1–79) in patients who did not develop kidney injury, 1.2 (0.2–63.3) in stage 1 patients, 2.1 (0.2–97,7) in stage 2 patients, and 4.4 (0.2–42.3) in stage 3 patients. According to the AKIN criteria, there was no significant difference between the patients who did not develop renal injury and those who developed stage 1 (*p* = 0.117) kidney injury when the stages of kidney injury were compared with the medians of the PIM-2 score. There was a significant difference between patients without kidney injury and patients with stage 2 (*p* = 0.001) and stage 3 (*p* = 0.001) kidney injury. There was no significant difference between stage 1 and 2 kidney injuries (*p* = 0.340), between stage 1 and 3 kidney injuries (*p* = 0.224), and between stage 2 and 3 kidney injuries (*p* = 0.610) (Figure 1D).

### 3.2. RIFLE Criteria

In terms of the age-related subgroup analysis, the pRIFLE criteria revealed no statistically significant difference between patients with and without kidney injury (*p* > 0.05). There was a statistically significant difference between the individuals with failure plus loss (*p* = 0.001) and those without kidney injury (*p* > 0.05). The risk and injury stages and risk and failure plus loss stages did not differ significantly (*p* > 0.05), but the risk and failure plus loss stages did differ significantly (*p* > 0.05), in terms of the length of PICU stay. According to the pRIFLE criteria, there was a significant difference in the length of PICU stay between patients with and without kidney injury (*p* = 0.001), injury (*p* = 0.001), and failure plus loss (*p* = 0.001). For the duration of PICU stay, there was no statistically significant difference between the risk and injury (*p* > 0.05), risk and failure plus loss (*p* > 0.05), or injury and failure plus loss (*p* > 0.05) stages.

**Figure 2:**
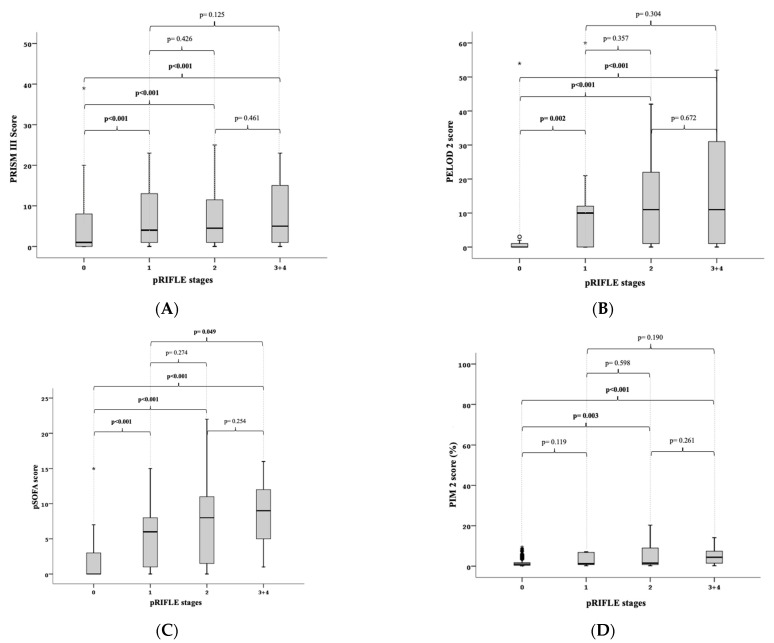
(**A**) pRIFLE stages and PRISM III scores: According to the pRIFLE criteria, there was no significant difference between the risk stage (*p* = 0.076) and patients without kidney injury when the stages of renal injury were compared with the medians of the PRISM III score. Patients with and without renal injury were significantly different from each other in the injury (*p* = 0.004) and failure plus loss (*p* = 0.008) stages. The stages of risk and injury (*p* = 0.878), risk and failure plus loss (*p* = 0.616), and injury and failure plus loss (*p* = 0.620) did not significantly differ from one another. (**B**) pRIFLE stages and PELOD 2 scores: According to the pRIFLE criteria, there was a significant difference between patients without kidney injury and patients with kidney injury in the risk (*p* = 0.002), injury (*p* < 0.001), and failure plus loss (*p* < 0.001) stages when the stages of renal injury were compared with the medians of the PELOD-2 score. The stages of risk and injury (*p* = 0.357), risk and failure plus loss (*p* = 0.304), and injury and failure plus loss (*p* = 0.672) did not significantly differ from one another. (**C**) pRIFLE stages and pSOFA scores: According to the pRIFLE criteria, there was a significant difference between patients without kidney injury and patients with kidney injury in the risk (*p* < 0.001), injury (*p* < 0.001), and failure plus loss (*p* < 0.001) stages when the medians of the kidney injury stages and the pSOFA score were compared. The risk and injury stages (*p* = 0.274) and the injury and failure plus loss stages (*p* = 0.254) did not significantly differ from one another, whereas the risk and failure plus loss stages (*p* = 0.049) did. (**D**) pRIFLE stages and PIM 2 scores: According to the pRIFLE criteria, there was no significant difference between patients without kidney injury and patients with kidney injury in the risk (*p* = 0.119) stage when the stages of renal injury were compared with the medians of the PIM-2 score. The difference between the patients with and without renal injury was statistically significant in both the injury (*p* = 0.003) and failure plus loss (*p* < 0.001) stages. The stages of risk and injury (*p* = 0.598), risk and failure plus loss (*p* = 0.190), and injury and failure plus loss (*p* = 0.261) did not significantly differ from one another.

When the PRISM III score was taken into account in the subgroup analysis, the median PRISM III score for patients without renal injury was 1 (0–39), while it was 4 (0–40), 4.5 (0–35), and 5 (0–41) for patients in the risk, injury, and failure plus loss stages, respectively. According to the pRIFLE criteria, there was no significant difference between the risk group (*p* = 0.076) and patients without kidney injury when the stages of renal injury were compared with the medians of the PRISM III score. Patients with and without renal injury were significantly different from each other in the injury (*p* = 0.004) and failure plus loss (*p* = 0.008) stages. The stages of risk and injury (*p* = 0.878), risk and failure plus loss (*p* = 0.616), and injury and failure plus loss (*p* = 0.620) did not significantly differ from one another (Figure 2A).

When the PELOD-2 score was taken into account in the subgroup analysis, the median PELOD-2 score for patients without kidney injury was 0 (0–54), while it was 10 (0–60) for the risk stage, 11 (0–42) for the injury stage, and 11 (0–52) for the failure plus loss stage. When the stages of kidney injury according to the pRIFLE criteria were compared with the medians of the PELOD 2 score, there was a significant difference between the patients without kidney injury and the risk (*p* = 0.002), injury (*p* < 0.001), and failure plus loss (*p* < 0.001) stages of kidney injury. However, the stages of risk and injury (*p* = 0.357), risk and failure plus loss (*p* = 0.304), and injury and failure plus loss (*p* = 0.672) did not significantly differ from one another (Figure 2B).

In individuals without kidney injury, the median pSOFA score was 0 (0–15), while it was 6 (0–15) in the risk stage, 8 (0–22) in the injury stage, and 9 (1–16) in the failure plus loss stage when taken into account in the subgroup analysis. According to the pRIFLE criteria, there was a significant difference between patients without kidney injury and patients with kidney injury in the risk (*p* < 0.001), injury (*p* < 0.001), and failure plus loss (*p* < 0.001) stages when the medians of the kidney injury stages and the pSOFA score were compared. The risk and injury stages (*p* = 0.274) and injury and failure plus loss stages (*p* = 0.254) did not significantly differ from one another, whereas the risk and failure plus loss stages (*p* = 0.049) did (Figure 2C).

The median PIM-2 score was found to be 1 (0.1–79) in patients without kidney injury, 1.2 (0.2–63.3) in the risk stage, 1.5 (0.2–97.7) in the injury stage, and 4.4 (0.2–42.3) in the failure plus loss stage when taken into account in the subgroup analysis. According to the pRIFLE criteria, there was no significant difference between patients without kidney injury and patients with kidney injury in the risk (*p* = 0.119) stage when the stages of renal injury were compared with the medians of the PIM-2 score. In both the injury (*p* = 0.003) and failure plus loss (*p* 0.001) stages, the difference between patients with and without renal injury was statistically significant. The stages of risk and injury (*p* = 0.598), risk and failure plus loss (*p* = 0.190), and injury and failure plus loss (*p* = 0.261) did not significantly differ from one another (Figure 2D).

The age, gender, and PIM-2 score variables were not observed to have any effect on renal injury in the logistic regression model analysis for kidney injury. It was found that kidney injury was significantly influenced by the number of diagnoses, the number of comorbidities, and carbapenem, aminoglycoside, penicillin, and quinolone use (Table 3). The occurrence of renal injury was found to be statistically strongly and significantly correlated with high PRISM III, PELOD-2, and pSOFA scores (Table 3).

## 4. Discussion

In this study, according to the pRIFLE and AKIN criteria, renal injury was seen in 8.7% of patients. AKI is frequent in hospitalized patients (8–30% in PICUs) and increases morbidity and mortality. Patients with AKI who have recently recovered are at risk of long-term renal complications. Identifying patients at risk and taking necessary precautions are important public health goals to prevent long-term complications of AKI [15,16]. It has been reported that the development of kidney injury is related to medications in approximately 25% of critically ill children [17,18,19]. Gowa et al.’s [20] study, which included 99 children in a PICU, reported that 97.9% of the patients were exposed to nephrotoxic drugs, and 46.5% of the patients developed drug-induced AKI. In our study, the rates of medications that are common in PICUs were found to be significantly higher in patients with AKI. The most commonly used antibiotic groups in patients who developed AKI were the beta-lactam and carbapenem groups, and the second most frequently used were the cephalosporin and glycopeptide groups.

In our study, younger age, the number of comorbidities, the development of kidney injury, and the use of carbapenem, cephalosporin, penicillin, and glycylcycline were found to have a significant effect on mortality, and it was also found that the pSOFA score was significantly associated with mortality. In our study, 47% of the children who died had AKI, while only 5% of the children who survived had AKI. In studies conducted in PICUs, varying mortality rates have been reported in patients who developed AKI. According to the pRIFLE criteria, Gupta et al. [21] reported a mortality rate of 65%, and Martin et al. [22] reported a mortality rate of 44% in patients who developed AKI. Using the AKIN criteria, Nawaz et al. [23] found that patients who acquired AKI had a mortality rate of 40%, whereas Saritha et al. [24] found that patients who developed AKI had a mortality rate of 24.7%. In our study, according to the AKIN criteria, the mortality rate was found to be 26.3% in patients diagnosed with stage 1 AKI, 50% in patients diagnosed with stage 2 AKI, and 66.7% in patients diagnosed with stage 3 AKI. According to the pRIFLE criteria, the mortality rate was found to be 29.4% in the risk stage, 46.4% in the injury stage, and 64.7% in patients who acquired AKI. Furthermore, according to the pRIFLE criteria, the mortality rate increased dramatically as the AKI stage advanced. The presence of advanced pRIFLE and AKIN stages was found to be associated with the mortality of a large proportion of individuals.

The occurrence of renal injury was found to be statistically strongly and significantly correlated with high PRISM III, PELOD-2, and pSOFA scores. When the stages of kidney injury according to the AKIN criteria were compared with the PRISM III, PELOD 2, and pSOFA scores, a significant difference was found between the patients who did not develop AKI and those who developed stage 1, stage 2, and stage 3 kidney injury. For the PRISM III, PELOD 2, and pSOFA scores, there were no significant differences between the stages according to the AKIN criteria. According to the AKIN criteria, 60.2% of the patients in a prospective study involving 108 patients in adult intensive care units were found to have AKI, and both the incidence of AKI and ICU mortality were higher among patients with a SOFA score above nine [25]. A substantial difference was discovered between the patients who did not develop AKI and those who were in the risk, injury, and failure plus loss stages when the pRIFLE criteria and pSOFA score were taken into account. The risk and failure plus loss stages showed a substantial difference; however, there was no discernible difference between the risk and injury, failure, or loss stages. In a retrospective study involving 543 adult patients in three intensive care units, it was found that the median SOFA score significantly increased as the RIFLE criteria stage climbed in patients who had AKI [26]. According to the pRIFLE criteria, patients with and without renal injury had significantly different PRISM III scores from each other in the injury and failure plus loss stages. In a retrospective analysis of 103 patients in a tertiary pediatric intensive care unit who acquired AKI in line with the pRIFLE criteria, the mean PRISM III scores for the non-AKI, risk, injury, and failure classes were found to be 3.45, 5.44, 6.92, and 8.92, respectively, and the PRISM III score was greater in patients with a more severe pRIFLE stage [27].

According to the PIM-2 ratio and the pRIFLE criteria, there was a statistically significant difference between patients in the injury and failure plus loss stages and those who did not develop AKI. In a study that looked at 126 patients in a pediatric intensive care unit, according to the pRIFLE criteria, patients who had AKI had higher median PIM-2 scores in escalating phases [28]. A substantial difference was discovered between the patients who did not develop AKI and those who were in the risk, injury, and failure plus loss stages when the pRIFLE criteria and PELOD-2 score were taken into account. Patients in the stages of risk and injury, risk and failure plus loss, and injury and failure plus loss did not significantly differ from one another. 

This study has some limitations. For instance, this was a retrospective, single-center study, and our center is a tertiary care PICU where patients with underlying diseases are more likely to be admitted.

## 5. Conclusions

Our study is the first pediatric study to demonstrate the relationship between the AKIN and pRIFLE AKI classifications and the PRISM III, PIM-2, PELOD-2, and pSOFA scores, which are commonly used in pediatric intensive care units to assess mortality risk in critically ill children without kidney disease. It is believed that identifying the risk factors for the development of AKI and planning antimicrobial regimens for patients with favorable prognoses at the time of intensive care unit admission could lower mortality rates. Further new studies including new parameters for the evaluation of kidney damage in intensive care units are needed.

## Figures and Tables

**Table 1:** Demographic and clinical factors, medications, and interventions in pediatric intensive care among children with or without acute kidney injury.

	Acute Kidney Injury		*p*
Variables	Exists (n = 62)	None (n = 654)	
Age (months)	21.5 (1–204)	50 (1–218)	0.004 ^c^
Gender (boys/girls)	40/22	330/324	0.034 ^a^
Mechanical ventilation	42 (67.77)	172 (26.3)	<0.001 ^a^
Invasive mechanical ventilation	41 (97.6)	123 (71.5)	<0.001 ^b^
Length of stay (days)	27.5 (1–746)	3 (0–250)	<0.001 ^c^
PRISM III score	5 (0–41)	1 (0–39)	<0.001 ^c^
PELOD 2 score	11 (0–60)	0 (0–54)	<0.001 ^c^
pSOFA score	7 (0–22)	0 (0–15)	<0.001 ^c^
PIM-2 score	1.8 (0.2–97.7)	1 (0.1–79)	<0.001 ^c^
Mortality	29 (46.8)	32 (4.9)	<0.001 ^c^
Antifungals	22 (35.5)	51 (7.8)	<0.001 ^a^
Antibiotics (n/%)	59 (95.2)	403 (61.6)	<0.001 ^b^
Carbapenem	43 (69.4)	118 (18)	<0.001 ^a^
Macrolide	15 (24.2)	74 (11.3)	0.003 ^a^
Glycopeptide	40 (64.5)	144 (22)	<0.001 ^a^
Beta-lactam	43 (69.4)	341 (52.1)	0.009 ^a^
Cephalosporin	40 (64.5)	315 (48.2)	0.014 ^a^
Penicillin	12 (19.4)	44 (6.7)	<0.001 ^a^
Colistin	16 (25.8)	25 (3.8)	<0.001 ^a^
Aminoglycoside	17 (27.4)	39 (6)	<0.001 ^a^
Glycylcycline	15 (24.2)	22 (3.4)	<0.001 ^a^
Quinolone	9 (14.5)	5 (0.8)	<0.001 ^a^
Lincosamide	5 (8.1)	6 (0.9)	<0.001 ^a^
Sulfonamide/cotrimoxazole	8 (12.9)	6 (0.9)	<0.001 ^a^
Metronidazole	6 (9.7)	31 (4.7)	0.093 ^a^
Anticonvulsives	23 (37.1)	157 (24)	0.023 ^a^
Steroids	31 (50)	149 (22.8)	<0.001 ^a^
Inotropes	42 (67.7)	84 (12.8)	<0.001 ^a^
PPI	34 (54.8)	173 (26.5)	<0.001 ^a^
Sedo-analgesia	28 (45.2)	153 (23.4)	<0.001 ^a^
Paracetamol	20 (32.3)	81 (12.4)	<0.001 ^a^
Ibuprofen	5 (8.1)	10 (1.5)	0.001 ^a^
Diuretics	27 (43.5)	43 (6.6)	<0.001 ^a^
Antihypertensives	21 (33.9)	37 (5.7)	<0.001 ^a^
Antiarrhythmics	13 (21)	43 (6.6)	<0.001 ^a^
Antipsychotics	0 (0)	3 (0.5)	1.0 ^b^
Vasopressin	4 (6.5)	1 (0.2)	<0.001 ^b^

^a^ Pearson chi-square test, ^b^ Fischer exact test, ^c^ Mann–Whitney U test.

**Table 2:** Comparison of the presence of acute kidney injury according to the pRIFLE and AKIN criteria and the presence of mechanical ventilation in critically ill children regarding prognosis.

Variables	Mortality		
	Exitus (n = 61)	Survival (n = 655)	*p*
Kidney injury	29 (47.5)	33 (5)	<0.001 ^a^
pRIFLE No injury Risk Injury Failure Loss	32 (52.5) 5 (8.2) 13 (21.3) 10 (16.4) 1 (1.6)	622 (95) 12 (1.8) 15 (2.3) 6 (0.9) 0 (0)	<0.001 ^a^
AKIN No injury Stage 1 Stage 2 Stage 3	32 (52.5) 5 (8.2) 14 (23) 10 (16.4)	622 (95) 14 (2.1) 14 (2.1) 5 (0.8)	<0.001 ^a^
Mechanical ventilation	59 (96.7)	155 (23.7)	<0.001 ^b^
Invasive mechanical ventilation	58 (98.3)	106 (68.4)	<0.001 ^b^
Age (months)	17 (1–174)	50 (1–218)	<0.001 ^c^
Presence of comorbidities	1 (0–4)	0 (0–4)	<0.001 ^c^
Length of stay (days)	23 (0–746)	3 (0–459)	<0.001 ^c^
PRISM III	11 (0–41)	1 (0–25)	<0.001 ^c^
PELOD 2	21 (0–60)	0 (0–41)	<0.001 ^c^
pSOFA	9 (1–22)	0 (0–14)	<0.001 ^c^
PIM 2	3.7 (0.1–97.7)	1.0 (0.1–79)	<0.001 ^c^

^a^ Chi-square test, ^b^ Fischer exact test, ^c^ Mann–Whitney U test.

**Table 3:** Logistic regression analysis of risk factors related to acute kidney injury.

Variables	B	SE	Exp (B)	*p* Value
Number of diagnoses	0.94	0.197	2.57	<0.001
Comorbidities	0.57	0.151	1.77	<0.001
Carbapenem	1.95	0.394	7.00	<0.001
Aminoglycoside	0.98	0.466	2.67	0.035
Penicillin	1.09	0.447	3.01	0.014
Quinolone	1.83	0.736	6.26	0.013
PRISM III	−0.76	0.027	0.93	0.004
PELOD 2	0.05	0.022	1.05	0.024
pSOFA	0.29	0.054	1.33	<0.001

## Data Availability

The data presented in this study are available on request from the corresponding author. The data are not publicly available due to privacy reasons.
